# Relationship between the stress–hyperglycemia ratio and atrial fibrillation recurrence risk after radiofrequency catheter ablation: a retrospective study

**DOI:** 10.3389/fcvm.2025.1699551

**Published:** 2025-12-02

**Authors:** Meng-li Li, Ning-Jun Zhu, Zhen Wang, Ting-ting Fan, Xiao-chen Wang, Xun Yang

**Affiliations:** 1 Department of Cardiology, The Second Affiliated Hospital of Anhui Medical University, Hefei, Anhui, China; 2 Department of Cardiothoracic Surgery, The First Affiliated Hospital of Anhui Medical University, Hefei, Anhui, China; 3 Department of Cardiothoracic Surgery, Anhui Public Health Clinical Center, Hefei, Anhui, China

**Keywords:** stress–hyperglycemia ratio, atrial fibrillation, radiofrequency catheterablation, atrial fibrillation recurrence, prognostic biomarker, nomogram

## Abstract

**Background:**

The stress hyperglycemia ratio (SHR) is linked to cardiovascular outcomes. However, its role in predicting atrial fibrillation (AF) recurrence after radiofrequency catheter ablation (RFCA) remains unclear. Therefore, this study investigated the SHR as a potential prognostic biomarker for post-RFCA AF recurrence.

**Methods:**

In this retrospective cohort study, 446 symptomatic non-valvular atrial fibrillation patients who underwent radiofrequency catheter ablation were followed for 12–26 months. The stress hyperglycemia ratio (SHR) was calculated as admission fasting blood glucose (mmol/L)/[1.59 × HbA1c (%)−2.59]. Patients were classified based on SHR levels. The primary endpoint was atrial fibrillation recurrence, and the secondary endpoints were cardiovascular events and a composite endpoint comprising relevant clinical outcomes.

**Results:**

AF recurrence occurred in 128 patients (28.7%). Patients in the recurrence group exhibited significantly higher SHR levels (*p* < 0.001). Receiver operating characteristic (ROC) analysis identified an optimal SHR cutoff value of 0.91 for predicting recurrence. Furthermore, even after multivariable adjustment for diabetes, alcohol consumption, antiarrhythmic drug use, SGLT2 inhibitor use, left atrial diameter (LAD), and uric acid levels, elevated SHR remained significantly associated with AF recurrence (HR: 3.379, 95% CI: 2.272–5.025, *P* < 0.001). ROC analysis demonstrated that SHR had superior predictive performance compared with other glycemic parameters, with an area under the curve (AUC) of 0.79 (95% CI: 0.74–0.84), yielding a sensitivity of 79.7% and a specificity of 68.4%. A prognostic nomogram incorporating six independent predictors was developed to estimate 1- and 2-year recurrence-free survival. Formal interaction tests indicated no significant effect modification by diabetes status (*P* for interaction = 0.432), with consistent SHR-associated recurrence risks observed in both diabetic and non-diabetic subgroups. Sensitivity analyses confirmed the robustness of these findings, as statistical significance was maintained after excluding patients with prior open-heart surgery and when modeling SHR as tertiles.

**Conclusions:**

The increase in SHR is significantly correlated with atrial fibrillation recurrence and composite events after RFCA. These findings support its potential clinical application value in improving risk stratification and prognostic assessment of this patient population.

## 1 Introduction

AF is the most common cardiac arrhythmia globally and has witnessed a sustained increase. Currently, it affects over 60 million people worldwide (1–3). This rise is associated with longer lifespans and lifestyle changes, and it corresponds to higher risks of stroke, heart failure, and all—cause mortality (4). RFCA has become the first—line treatment for drug—resistant AF, restoring sinus rhythm by electrically isolating the pulmonary veins (5, 6). Despite technological advancements, postoperative AF recurrence remains high, with approximately 30%–40% of patients experiencing relapse within one year, which is attributed to multiple factors, including atrial structural remodeling and inflammatory responses (6, 7). Identifying biomarkers to guide intervention and optimize postoperative risk stratification and management has become a key focus of current clinical research.

Accumulating evidence from preliminary studies has implicated metabolic disturbances, especially hyperglycemia, as a critical factor in post-RFCA AF recurrence (8–10). Elevated blood glucose on admission, which often reflects stress hyperglycemia, serves as an independent predictor of adverse prognosis in various cardiovascular conditions, including heart failure, myocardial infarction, and arrhythmias (11, 12). However, this measure lacks specificity, as it may be influenced by either chronic hyperglycemia or an acute physiological stress response, which precludes its utility in precisely evaluating the acute dysmetabolic state.

Stress hyperglycemia at admission may originate from either chronic hyperglycemia or an acute physiological stress response, limiting its ability to accurately reflect true acute dysglycemia. In contrast, the stress hyperglycemia ratio (SHR)—a novel metric integrating acute admission blood glucose with chronic glycemic levels (as reflected by glycosylated hemoglobin)—can more effectively evaluate genuine acute hyperglycemic status, independent of pre-existing diabetes (13, 14). The clinical value of SHR is well established in the literature, where it has been associated with critical outcomes such as thrombotic burden, coronary artery disease severity, post-stroke cerebral edema, and hospital-acquired infection risk (12). Moreover, elevated SHR has demonstrated prognostic utility in predicting clinical outcomes, showing a significant correlation with 1-year all-cause mortality in both American and Chinese cohort studies (15, 16).

Despite this, the role of SHR in predicting outcomes following atrial fibrillation ablation remains systematically underexplored, as existing studies have primarily focused on conventional diabetic indicators while overlooking the independent prognostic impact of acute glycemic abnormalities.

Our study's specific objectives include (1) quantifying the association between the stress hyperglycemia ratio (SHR) and postoperative atrial fibrillation (AF) recurrence, defined as the recurrence of AF three months after ablation, and (2) evaluating the predictive value of the SHR for major cardiovascular events, including stroke, heart failure hospitalizations, and cardiovascular deaths. The research results are expected to provide new evidence for personalized interventions during the perioperative period, such as blood glucose control strategies, and to improve the precision of AF management.

## 2 Methods

### 2.1 Study design and population

This retrospective study consecutively enrolled 501 symptomatic patients with nonvalvular atrial fibrillation who underwent radiofrequency catheter ablation (RFCA) at our center between January 2023 and September 2024. The study aimed to include all eligible patients treated during this period.The inclusion criteria were: (1) aged 18–80 years; (2) met the ESC diagnostic criteria for nonvalvular AF; and (3) availability of complete follow-up data for at least 12 months post-procedure. Patients were excluded if they met any of the following criteria: (1) prior open-heart surgery; (2) life-threatening comorbidities, including active infectious diseases or severe hepatic/ renal dysfunction. Severe hepatic insufficiency was defined as liver cirrhosis (Child-Pugh class B or C) or baseline aminotransferase levels exceeding three times the upper limit of normal. Severe renal insufficiency was defined as an estimated glomerular filtration rate < 30 mL/min/1.73 m² (consistent with chronic kidney disease stages 4–5) or a requirement for renal replacement therapy (dialysis). (3) missing crucial baseline data required for analysis (e.g., HbA1c, fasting blood glucose). After applying these criteria, 55 patients were excluded. The specific reasons for exclusion were: [missing HbA1c or glucose data (*n* = 45), presence of life-threatening comorbidities (*n* = 10)]. Consequently, the final analysis cohort comprised 434 patients.

This study was conducted in accordance with the ethical principles of the Declaration of Helsinki and was approved by the Ethics Committee of the Second Affiliated Hospital of Anhui Medical University (Approval No: YX2024-232). Written informed consent was obtained from all participants.

### 2.2 Ablation procedure

This study exclusively enrolled patients who underwent radiofrequency catheter ablation (RFCA); those treated with cryoballoon ablation (CRYO) were excluded. Preprocedural data on antiarrhythmic drug (AAD) use and prior cardioversion history were systematically collected for all participants. All RFCA procedures were performed under conscious sedation. Following transseptal puncture, an initial heparin bolus (100 IU/kg) was administered, with subsequent continuous infusion to maintain an activated clotting time (ACT) between 300 and 350 s. The procedural cornerstone was extensive pulmonary vein isolation (PVI), performed using a three-dimensional electroanatomical mapping system (CARTO, Biosense Webster, or Ensite, Abbott).Continuous and contiguous point-by-point lesions were created around the ipsilateral pulmonary veins to establish a wide-area circumferential ablation line. The endpoint was successful electrical isolation of all pulmonary veins, confirmed by the absence or dissociation of PV potentials using a circular mapping catheter. This endpoint was achieved in all enrolled patients.

Ablation for non-pulmonary vein (non-PV) targets was performed at the operator's discretion based on clinical and electrophysiological findings. In patients with documented or inducible typical atrial flutter, a cavotricuspid isthmus (CTI) ablation line was created to achieve bidirectional conduction block. For patients with organized atrial tachycardias (AT) or suspected non-PV triggers, further mapping and ablation were conducted. If sustained AF persisted after PVI, electrical cardioversion was applied to restore sinus rhythm.

### 2.3 Data collection

Data were extracted from the hospital's electronic medical record system. Sociodemographic details included age, sex, body mass index, smoking status, and alcohol consumption-the latter quantified as significant intake (>210 g/week for men and >140 g/week for women). Medical history encompassed hypertension, diabetes, coronary artery disease, cerebral apoplexy, chronic obstructive pulmonary disease, atrial fibrillation (AF) type, AF duration, and the operating physician. Medication history was also recorded. Laboratory parameters, measured from venous blood samples collected after at least 8 h of fasting within 24 h of admission, included admission blood glucose (ABG), glycated hemoglobin (HbA1c), creatinine, uric acid, hemoglobin, total cholesterol, triglycerides, high-density lipoprotein cholesterol, low-density lipoprotein cholesterol, and thyroid-stimulating hormone. Echocardiographic measures comprised left atrial diameter (LAD), left ventricular end-diastolic diameter (LVEDD), interventricular septal thickness (IVS), left ventricular posterior wall thickness (LVPW), and left ventricular ejection fraction (LVEF). Procedural success, defined as complete electrical isolation, was confirmed in all patients.

### 2.4 Assessment of SHR

SHR was calculated using the formula: SHR = admission fasting blood glucose (mmol/L)/[1.59 × HbA1c (%)−2.59]. To determine the optimal cutoff value of SHR for predicting AF recurrence, we performed a Receiver Operating Characteristic (ROC) curve analysis. The optimal cutoff point was defined as the SHR value that maximized the Youden index (J = sensitivity + specificity −1). This analysis yielded an optimal SHR cutoff value of 0.91. Based on this cutoff, the study population was stratified into two groups: a high-SHR group (SHR ≥ 0.91, *n* = 181) and a low-SHR group (SHR < 0.91, *n* = 265).

### 2.5 Follow-up and outcomes

All enrolled patients were actively followed for a minimum of 12 months, with data collection ongoing until September 2025. The primary outcome was the recurrence of atrial fibrillation (AF), defined as any atrial tachyarrhythmia (including AF, atrial flutter, or atrial tachycardia) lasting for ≥30 s, as documented on electrocardiographic monitoring after a 3-month blanking period following the ablation procedure. The methodology for detecting AF recurrence was designed to capture both symptomatic and asymptomatic episodes. It consisted of a structured protocol (1) Scheduled Interrogations: All patients underwent systematic rhythm assessment at pre-specified outpatient visits at 3, 6, and 12 months post-ablation. This included a 12-lead electrocardiogram (ECG) and a 24 h ambulatory Holter monitor. (2) Symptom-Driven Recordings: Patients were provided with handheld single-lead ECG devices and instructed to record and transmit a tracing whenever they experienced palpitations or other arrhythmia-related symptoms. (3) Adjudication of Events: All recorded electrocardiographic tracings—whether from our institution, external hospitals, or patient-transmitted data—were reviewed and confirmed by two independent cardiologists who were blinded to the patients' SHR group assignment. Any discrepancy was resolved by a third senior electrophysiologist.

Secondary outcomes included other cardiovascular events (such as hospitalization for heart failure, acute myocardial infarction, cardiovascular death, and significant procedural complications like cardiac tamponade or pulmonary vein stenosis) and non-cardiovascular events (including stroke, major bleeding, and non-cardiac hospitalization or death).

### 2.6 Statistical analysis

Data analysis was performed using SPSS 26.0 (IBM Corp., Armonk, NY, USA) and R software (version 4.3.1; R Foundation for Statistical Computing, Vienna, Austria). A two-sided *P*-value < 0.05 was defined as statistically significant.Given the retrospective nature of this study, cases with any missing data for the analyzed variables were excluded from the final analysis to ensure data integrity. Continuous variables were assessed for normality using the Shapiro–Wilk test. Based on this assessment, normally distributed data are presented as means ± standard deviations and were compared between the two SHR groups using the independent samples *t*-test. Non-normally distributed data are presented as medians [interquartile ranges] and were compared using the Mann–Whitney *U* test. Categorical variables are expressed as numbers (percentages) and were compared using the Chi-square test, or Fisher's exact test, as appropriate. Time-to-event data for atrial fibrillation recurrence were analyzed using Kaplan–Meier survival curves. The log-rank test was used to compare recurrence-free survival between the two SHR groups. The association of variables with AF recurrence was quantified using the Cox proportional hazards model. Results are reported as Hazard Ratios (HRs), with 95% confidence intervals (CIs).

Variables showing a univariate association with the outcome at a significance level of *P* < 0.1 were considered candidates for inclusion in the multivariate Cox model. This relaxed threshold of *P* < 0.1 is a conventional method to minimize the omission of potential confounding variables from the final model. The final multivariate model was constructed to identify independent factors associated with AF recurrence, adjusting for these candidate variables.

### 2.7 Subgroup and sensitivity analyses

To assess the consistency and robustness of our primary findings, we conducted pre-specified subgroup and sensitivity analyses. A subgroup analysis was performed based on diabetes status. The presence of an effect modification by diabetes was formally tested by introducing an interaction term (SHR × diabetes status) into the multivariate Cox proportional hazards model. A two-sided *P* value for interactio*n* < 0.05 was considered statistically significant. Additionally, we performed two sensitivity analyses: first, by repeating the primary analysis after excluding five patients with a history of open-heart surgery to address potential confounding from a profoundly altered atrial substrate; and second, by reclassifying SHR based on tertiles to examine if the results were dependent on the specific binary cut-off value.

## 3 Results

### 3.1 Baseline characteristics according to atrial fibrillation recurrence Status

The final analysis included 446 patients with atrial fibrillation. Comparative assessment of baseline characteristics between the recurrence (*n* = 128) and nonrecurrence (*n* = 318) groups revealed statistically significant differences in several parameters, as summarized in Table 1.

**Table 1:** Baseline characteristics of patients with atrial fibrillation in the recurrence and non-recurrence groups.

Baseline variables	Total	Recurrence-free group	Recurrence group	*P* value
	*N* = 446	(*n* = 318)	(*n* = 128)	
Age (years)	66 (58, 72)	66 (59, 71)	67 (58, 72)	0.996
Female, *n* (%)	187 (41.9)	133 (41.8)	54 (42.2)	0.944
BMI	25.1 (23.2, 27.1)	25.1 (23.4, 27.4)	25 (22.5, 26.6)	0.220
Comorbidities, *n* (%)				
CAD	87 (19.5)	58 (18.2)	29 (22.7)	0.286
Hypertension	229 (51.3)	165 (52.4)	65 (48.9)	0.718
DM	171 (38.3)	111 (34.9)	60 (46.9)	0.019
CI	82 (18.4)	57 (17.9)	25 (19.5)	0.691
COPD	51 (11.4)	35 (11)	16 (12.5)	0.654
Smoking	118 (26.5)	83 (26.1)	35 (27.3)	0.787
Alcohol	302 (67.7)	208 (65.4)	98 (76.6)	0.065
AF type				0.956
Paroxysmal	203 (45.5)	145 (45.6)	58 (45.3)	
Persistent	243 (54.5)	173 (54.4)	70 (54.7)	
AF duration, months	18 (12, 36)	18 (12, 36)	24 (12, 48)	0.661
Laboratory data				
HbA1c (%)	5.9 (5.6, 6.5)	5.9 (5.6, 6.4)	6 (5.7, 6.8)	0.002
ABG (µmol/L)	5.7 (4.9, 7.2)	5.4 (4.8, 6.3)	7.1 (5.6, 8.9)	<0.001
Hemoglobin, g/dL	136 (125, 148)	137 (126, 150)	134 (122, 146)	0.341
TSH (mg/L)	2.4 (1.4, 3.7)	2.4 (1.5, 3.6)	2.3 (1.4, 3.9)	0.468
TC(mmol/L)	4.1 (3.5, 4.7)	4.1 (3.5, 4.7)	4.0 (3.4, 4.7)	0.921
TG(mmol/L)	1.3 (1.0, 1.7)	1.3 (0.9, 1.7)	1.3 (0.9, 1.7)	0.423
LDL-C(mmol/L)	2.5 (1.9, 3.0)	2.5 (1.9, 3.1)	2.4 (1.9, 2.9)	0.741
HDL-C(mmol/L)	1.1 (0.9, 1.3)	1.1 (0.9, 1.3)	1.1 (0.9, 1.3)	0.745
Creatinine(umol/L)	72 (61, 84)	73 (62, 86)	69 (60, 80)	0.612
UA(umol/L)	371 (306, 434)	359 (290, 432)	381 (326, 4,527)	0.041
SHR	0.9 (0.7, 1.0)	0.8 (0.7, 0.9)	1.0 (0.9, 1.1)	<0.001
SHR ≥ 0.91	181 (40.6)	98 (30.8)	83 (64.8)	<0.001
Medication data				
ACEI + ARB	121 (27.1)	87 (27.4)	34 (26.6)	0.864
Calcium channel blockers	104 (23.3)	76 (23.9)	28 (21.9)	0.997
Beta-blockers	76 (17)	56 (17.6)	20 (15.6)	0.613
SGL-2 inhibitor	125 (28)	98 (30.8)	27 (21.1)	0.039
Statins	212 (47.5)	150 (47.2)	62 (48.4)	0.808
Anticoagulants	429 (96.2)	307 (96.5)	122 (95.3)	0.734
Antiarrhythmics	363 (81.4)	269 (84.6)	94 (73.4)	0.006
Echocardiography				
LVEDD (mm)	47 (44, 51)	47 (44, 50)	48 (44, 52)	0.184
LAD	41 (36, 46)	40 (35, 44)	44 (39, 48)	<0.001
LVEF (%)	62 (59, 64)	62(59, 64)	62(60, 64)	0.478
IVS	10(9, 11)	10(9, 11)	10(9, 11)	0.212
LVPW	9(8, 10)	9(8, 10)	9(8, 10)	0.771

CAD, coronary artery disease; DM, diabetes mellitus; CI, cerebral infarction; COPD, chronic obstructive pulmonary disease; BMI, body mass index; HbA1c, glycated hemoglobin A1c; TC, total cholesterol; TG, triglycerides; HDL-C, high-density lipoprotein cholesterol; LDL-C, low-density lipoprotein cholesterol; UA, uric acid; Cr, creatinine; LAD, left atrial diameter; LVEDD, left ventricular end diastolic diameter; IVS, interventricular septal thickness; LVPW, left posterior wall thickness; LVEF, left ventricular ejection fraction; SHR, stress hyperglycemia ratio; ACEI + ARB, angiotensin-converting enzyme inhibitors/angiotensin receptor blockers; ABG, admission blood glucose; TSH, thyroid stimulating hormone.

Patients in the recurrence group showed higher prevalence of diabetes mellitus, higher HbA1c, higher admission blood glucose, and higher SHR. The recurrent group had a larger left atrial diameter and higher serum uric acid levels. Regarding drug use, the recurrence group had a lower utilization rate of antiarrhythmic drugs and SGLT-2 inhibitors. There were no significant differences between the two groups in terms of age, gender distribution, other comorbidities, blood lipids, ventricular function parameters, or the use of most cardiovascular drugs (see Table 1 for details).

### 3.2 Baseline characteristics determined based on the optimal critical value of stress hyperglycemia ratio

Comparative analysis showed that patients with SHR ≥ 0.91 had significantly higher prevalence of diabetes, larger left atrial diameter (LAD) and left ventricular end diastolic diameter (LVEDD), higher uric acid level, and increased statin use. Importantly, the high SHR group had a significantly higher recurrence rate of atrial fibrillation (see Table 2 for details).

**Table 2:** Baseline characteristics according to the best cutoff value for the stress hyperglycemia ratio.

Baseline variables	All patients	SHR < 0.91	SHR ≥ 0.91	*P* value
	*N* = (446)	*N* = 265	*N* = 181	
Age (years)	66 (58, 72)	67 (59, 73)	66 (57, 71)	0.979
Female, *n* (%)	187 (49.1)	115 (43.3)	72 (39.8)	0.447
BMI	24.9 (23.1, 27.2)	24.9 (23.4, 27.2)	25.2 (22.9, 21.2)	0.845
Comorbidities, *n* (%)				
CHD	86 (19.3)	47 (17.8)	39 (21.5)	0.326
Hypertension	229 (51.3)	136 (51.3)	93 (51.4)	0.99
Diabetes	171 (38.3)	76 (28.7)	95 (52.5)	<0.001
CI	82 (18.2)	47 (17.8)	34 (18.8)	0.792
COPD	50 (11.2)	25 (9.4)	25 (13.9)	0.144
Smoking	118 (26.5)	63 (23.8)	55 (30.4)	0.12
Alcohol	144 (32.3)	78 (29.4)	66 (36.5)	0.119
AF type				0.283
Paroxysmal	213 (47.8)	121 (45.7)	92 (50.8)	
Persistent	233 (52.5)	144 (54.3)	89 (49.2)	
AF duration, months	18 (12, 36)	18 (12, 36)	21 (12, 36)	0.102
Laboratory data				
Hemoglobin, g/dL	136 (126, 148)	136 (126, 148)	136 (123, 148)	0.528
TSH (mg/L)	2.4 (1.5, 3.7)	2.5 (1.9, 3.0)	2.5 (1.9, 3.0)	0.258
TC (mmol/L)	4.1 (3.5, 4.7)	4.1 (3.5, 4.7)	4.1 (3.5, 4.7)	0.465
TG (mmol/L)	1.3 (1.0, 1.7)	1.3 (1.0, 1.6)	1.4 (1.0, 1.7)	0.091
LDL-C (mmol/L)	2.5 (1.9, 3.0)	2.5 (1.9, 3.0)	2.5 (1.9, 2.9)	0.401
HDL-C (mmol/L)	1.1 (0.9, 1.2)	1.1 (1.0, 1.3)	1.0 (0.9, 1.3)	0.180
Creatinine (umol/L)	72 (61, 84)	72 (64, 84)	71 (61, 84)	0.065
UA (umol/L)	371 (306, 434)	361 (290, 429)	388 (332, 446)	0.004
Medication data				
ACEI + ARB	121 (27.1)	66 (24.9)	55 (30.4)	0.201
Calcium channel blockers	104 (23.3)	66 (24.9)	38 (21.0)	0.337
Beta-blockers	76 (17)	46 (17.4)	30 (16.6)	0.829
SGL-2	125 (22.6)	71 (26.8)	54 (29.8)	0.482
Statins	212 (47.5)	114 (43.0)	98 (54.1)	0.021
Anticoagulants	438 (98.2)	263 (99.2)	175 (96.7)	0.052
Antiarrhythmics	363 (81.34)	217 (81.9)	146 (80.7)	0.744
Echocardiography				
LVEDD (mm)	47 (44, 51)	47 (44, 50)	48 (44, 52)	0.031
LVEF (%)	62 (60, 64)	62 (60, 64)	62 (60, 64)	0.285
LA	41(36, 46)	40(35, 44)	44(37, 48)	<0.001
AF recurrence	133(29.8)	36(13.6)	97(53.6)	<0.001

CAD, coronary artery disease; DM, diabetes mellitus; CI, cerebral infarction; COPD, chronic obstructive pulmonary disease; BMI, body mass index; TC, total cholesterol; TG, triglycerides; HDL-C, high-density lipoprotein cholesterol; LDL-C, low-density lipoprotein cholesterol; UA, uric acid; Cr, creatinine; LAD, left atrial diameter; LVEDD, left ventricular end diastolic diameter; IVS, interventricular septal thickness; LVPW, left posterior wall thickness; LVEF, left ventricular ejection fraction; SHR, stress hyperglycemia ratio; ACEI + ARB, angiotensin-converting enzyme inhibitors/angiotensin receptor blockers; ABG, admission blood glucose; TSH, thyroid stimulating hormone; AF, atrial fibrillation.

### 3.3 Independent predictive factors of atrial fibrillation recurrence

After adjusting for potential confounding factors, multivariate Cox regression analysis identified several factors independently associated with the risk of atrial fibrillation recurrence. Elevated SHR (≥0.91) is the strongest predictor and significantly correlated with recurrence (adjusted HR = 3.379, 95% CI: 2.272–5.052). A history of alcohol consumption and non use of antiarrhythmic drugs are also significantly associated with a higher risk of recurrence. In continuous variables, higher levels of HbA1c, LAD, and uric acid are independently associated with increased risk. In contrast, the use of SGLT-2 inhibitors is significantly associated with a reduced risk of recurrence. It is worth noting that the history of diabetes is important in univariate analysis, but it is not an independent predictor after multivariate adjustment (see Table 3 for detailed data).

**Table 3:** Univariate and multivariate Cox regression analyses for clinical events.

Characteristics	HR(95% CI) Univariate analysis	*P* value Univariate analysis	HR(95% CI) Multivariate analysis	*P* value Multivariate analysis
Sex				
Male	Reference			
Female	0.943 (0.664–1.340)	0.745		
Age	0.996 (0.980–1.014)	0.682		
Alcohol				
NO	Reference		Reference	
YES	1.381 (0.933–2.044)	0.060	1.846 (1.213–2.811)	0.004
Statins				
YES	Reference			
NO	0.943 (0.667–1.334)	0.741		
Antiarrhythmics				
YES	Reference		Reference	
NO	1.698 (1.147–2.513)	0.008	1.577 (1.055–2.358)	0.026
AF type				
Persistent	Reference			
Paroxysmal	0.982 (0.694–1.390)	0.919		
AF duration time	1.036 (0.975–1.100)	0.251		
SGL-2 inhibitor				
NO	Reference			
YES	0.694 (0.454–1.062)	0.093	0.598 (0.372–0.961)	0.034
SHR ≥ 0.91				
NO	Reference		Reference	
YES	3.827 (2.657–5.511	< 0.001	3.379 (2.272–5.025)	< 0.001
LAD(mm)	1.065 (1.038–1.094)	< 0.001	1.034 (1.006–1.062)	0.018
LVEDD(mm)	1.026 (0.998–1.054)	0.069	1.001 (0.975–1.029)	0.916
Operators	0.962 (0.863–1.072)	0.480		
HbA1c (%)	1.539 (1.320–1.795)	< 0.001	1.454 (1.151–1.837)	0.002
BMI	0.967 (0.918–1.018)	0.203		
Hb(g/dL)	0.996 (0.986–1.003)	0.120		
UA(umol/L)	1.003 (1.002–1.005)	0.017	1.004 (1.002–1.005)	< 0.001
TSH (mg/L)	1.0094 (0.974–1.035)	0.798		
COPD				
YES	Reference			
NO	0.706 (0.439–1.137)	0.152		
Anticoagulants				
YES	Reference			
NO	1.405 (0.618–3.194)	0.417		
DM				
NO	Reference		Reference	
YES	1.689 (1.193–2.392)	0.003	0.790 (0.459–1.3460)	0.395

DM, diabetes mellitus; BMI, body mass index; UA, uric acid; Cr, creatinine; LAD, left atrial diameter; LVEDD, left ventricular end diastolic diameter; SHR, stress hyperglycemia ratio; TSH, thyroid stimulating hormone; AF, atrial fibrillation; HbA1c, glycated hemoglobin A1c; COPD, chronic obstructive pulmonary disease, Hb, hemoglobin.

### 3.4 The correlation between stress hyperglycemia ratio and clinical outcomes

**Figure 1:**
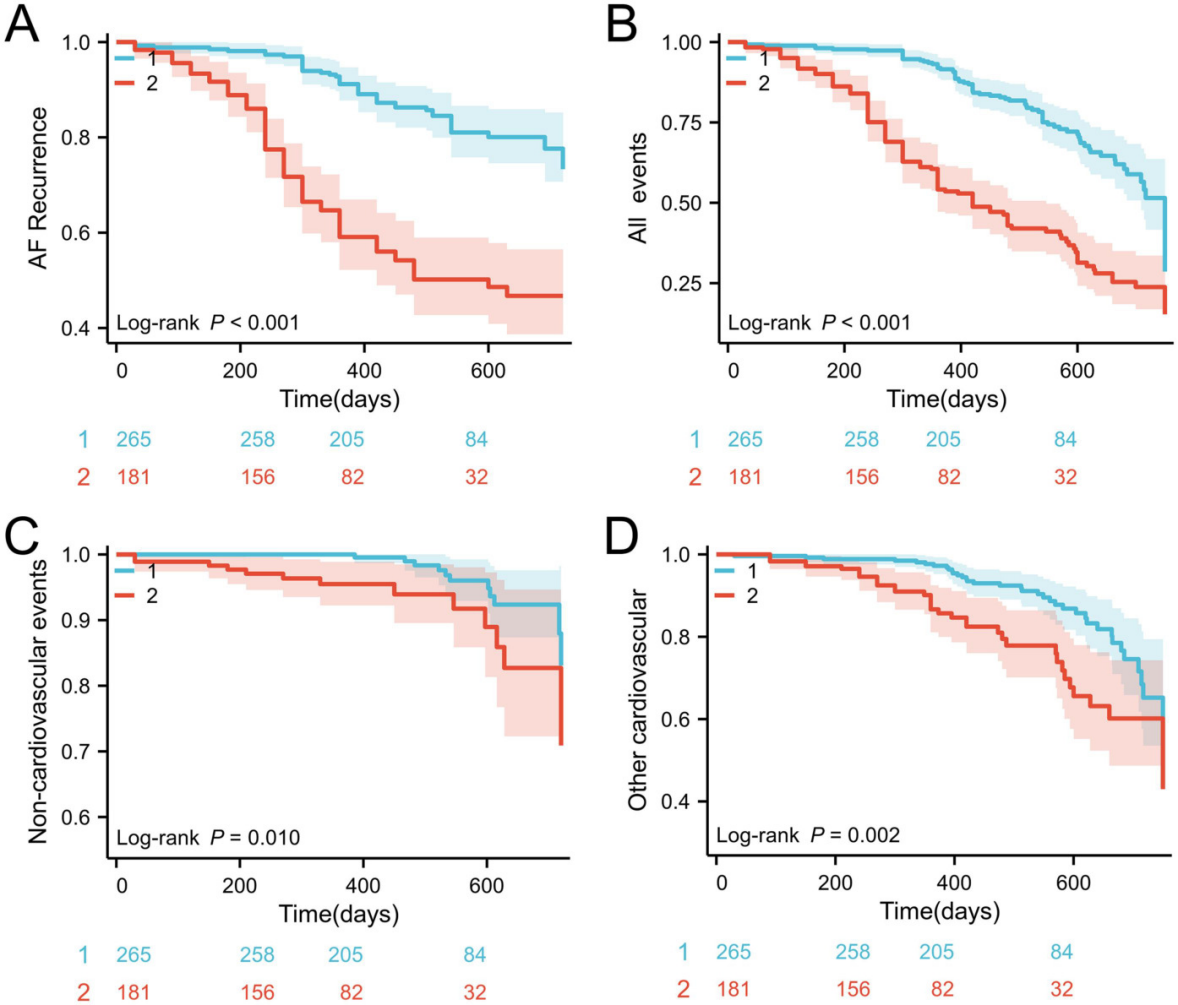
**(A)** Kaplan–Meier survival curves for AF recurrence events stratified by the SHR cutoff; **(B)** incidence of all events; **(C)** incidence of noncardiovascular mortality; **(D)** incidence of cardiovascular mortality. SHR, stress hyperglycemia ratio; AF, atrial fibrillation.

Consistent with preliminary analysis, the cumulative incidence of composite endpoints in the high SHR group was significantly higher, mainly due to atrial fibrillation recurrence. Kaplan Meier analysis confirmed this association, which showed that patients with SHR ≥ 0.91 had a significantly reduced probability of recurrence free survival (log rank *P* < 0.001); Figure 1A). Similarly, evaluations of secondary endpoints (including cardiovascular, non cardiovascular, and all-cause mortality) consistently showed poorer prognosis in the high SHR group (Figures 1B–D).

### 3.5 The predictive value of stress hyperglycemia ratio for atrial fibrillation recurrence

**Figure 2:**
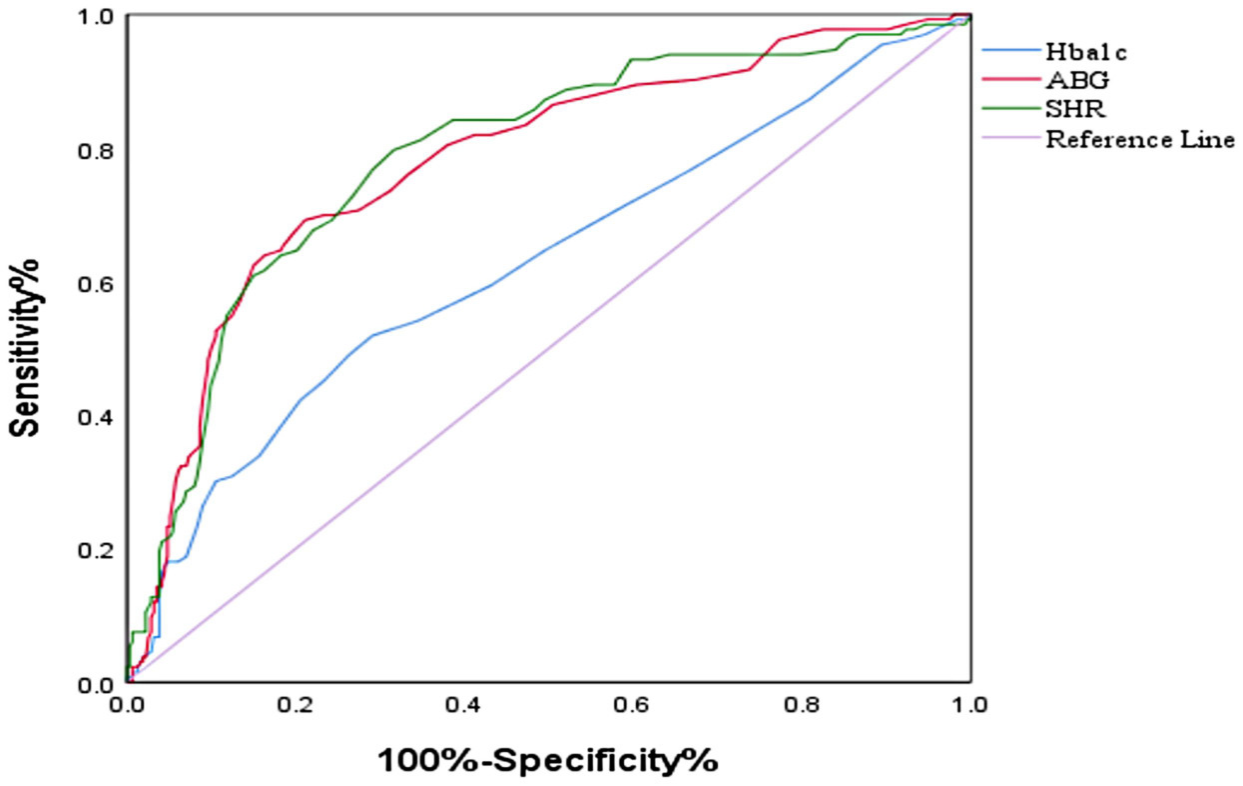
ROC curves of the SHR, HbA1c level and ABG for the prediction of AF recurrence. ROC, receiver operating curve; SHR, stress–hyperglycemia ratio; ABG, admission blood glucose; HbA1c, glycated hemoglobin.

ROC curve analysis is used to evaluate the diagnostic performance of HbA1c, ABG, and SHR in predicting outcomes. SHR has the best predictive performance, with specific AUC(0.79, 95% CI: 0.74–0.84) values shown in Figure 2.

### 3.6 Prediction model for atrial fibrillation recurrence

**Figure 3:**
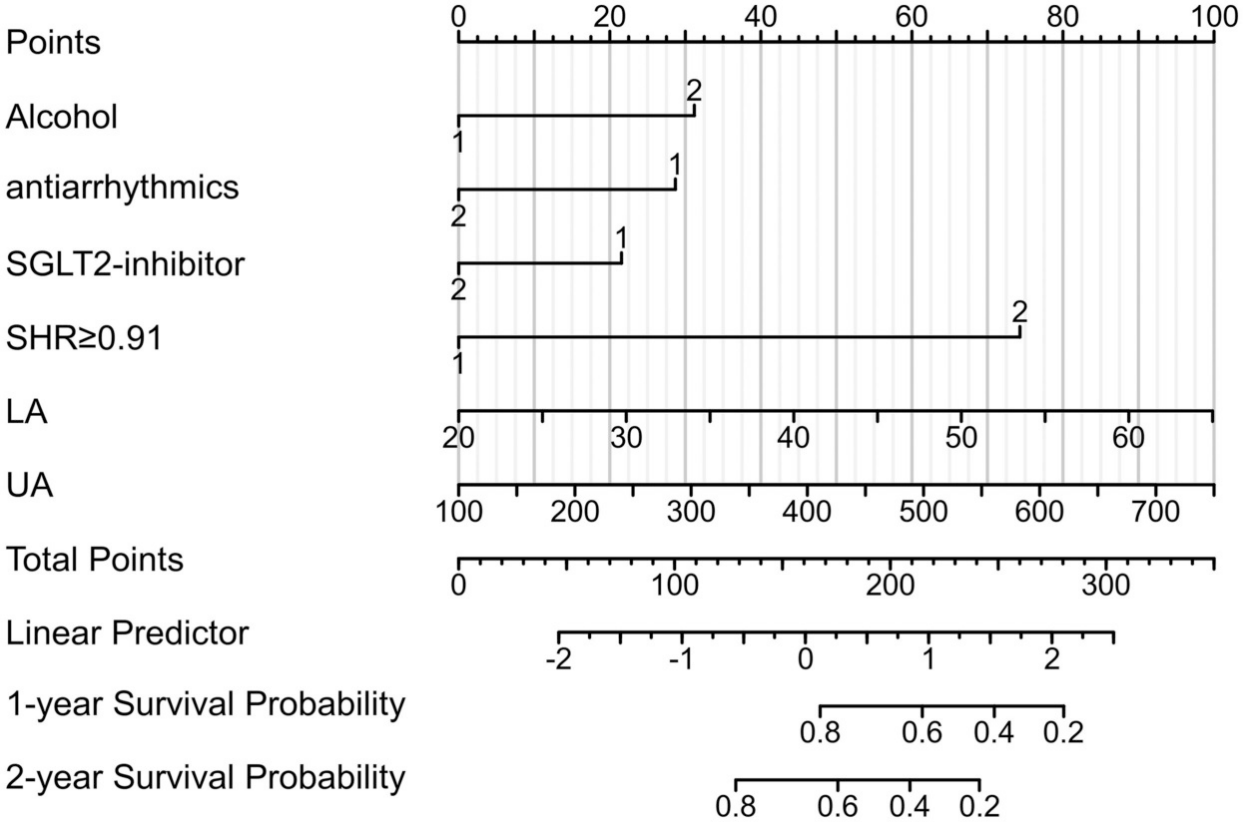
Nomogram of recurrence after RFCA for AF. UA, uric acid; LA, left atrial diameter; SHR, stress–hyperglycemia ratio.

Based on multivariate Cox regression analysis, a prognostic column chart was constructed to predict the 1-year and 2-year survival probabilities (Figure 3). The column chart includes six independent predictive factors: alcohol consumption; SHR; UA; LAD; And the use of antiarrhythmic drugs and SGL-2 inhibitors.

### 3.7 Subgroup and sensitivity analyses

**Figure 4:**
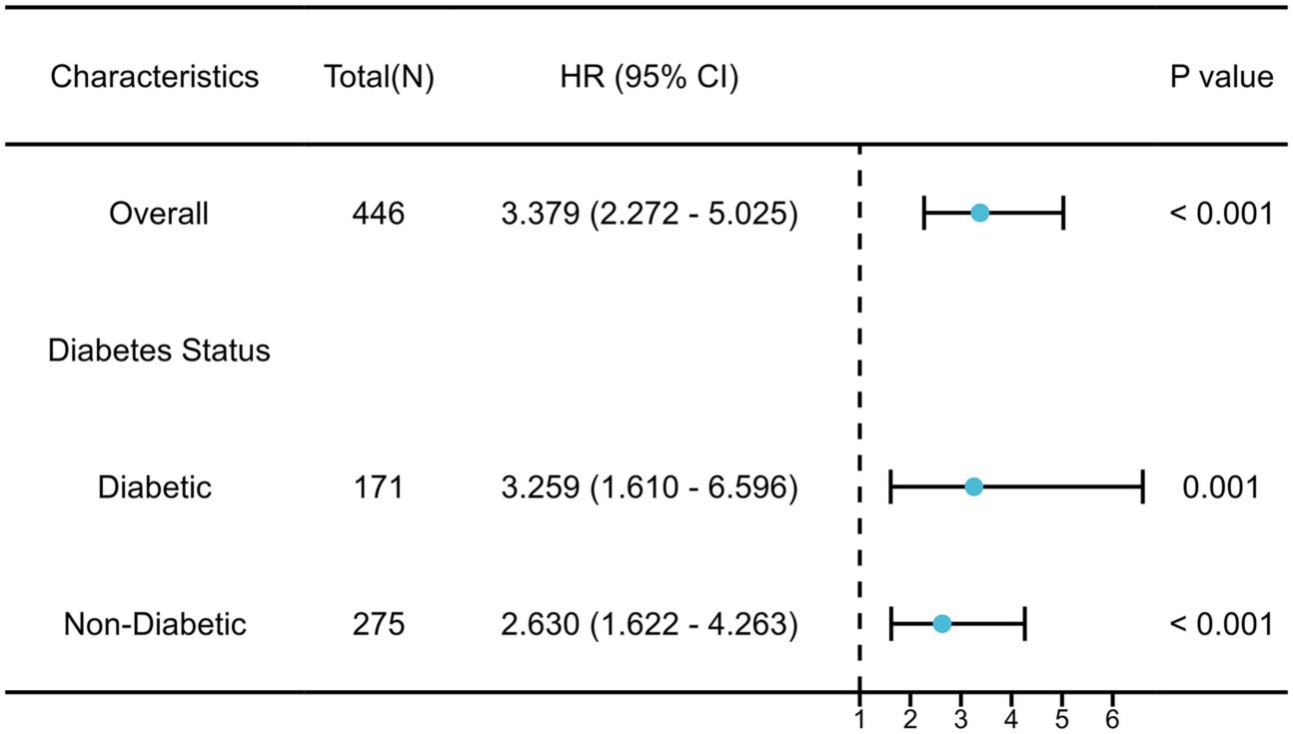
Subgroup analysis of the association between SHR and AF recurrence.

Formal tests of the interaction showed that diabetes status had no significant effect (interaction *P* = 0.432). As shown in the forest diagram (Figure 4), the elevation of SHR is consistent with the recurrence of atrial fibrillation in both diabetes patients and non diabetes patients. In all pre specified sensitivity analyses, the main finding remains robust. After excluding patients with a history of open heart surgery, the adjusted hazard ratio (HR) of SHR remained significant (adjusted HR = 3.451, 95% CI: 2.311–5.154). In addition, A significant graded association was observed across SHR tertiles. In the multivariable model, the highest SHR tertile was associated with a more than three-fold increased hazard of recurrence relative to the lowest tertile (adjusted HR = 3.38, 95% CI: 2.27–5.05). The detailed results of sensitivity analysis are shown in Supplementary Tables S1, S2.

## 4 Discussion

This study revealed a significant association between stress-induced hyperglycemia (SHR) and atrial fibrillation recurrence after radiofrequency catheter ablation (RFCA). This discovery has significant clinical implications as it elucidates for the first time that acute changes in metabolic status prior to ablation may have far-reaching effects on long-term surgical outcomes. In multivariate analysis, SHR ≥ 0.91 was significantly associated with recurrence risk, and this association remained robust in various sensitivity analyses, indicating that SHR may represent a novel and clinically feasible risk stratification tool. Compared with traditional single blood glucose or glycated hemoglobin measurements, SHR provides a more comprehensive assessment of patients' metabolic status by integrating acute and chronic blood glucose indicators, thereby enhancing the risk assessment ability for patients with complex arrhythmias. This discovery opens up new avenues for individualized treatment of atrial fibrillation, particularly for optimizing treatment strategies for patients with metabolic abnormalities.

Our findings provide a meaningful comparison and supplement to previous studies on the relationship between metabolic disorders and the prognosis of atrial fibrillation. Mohanty et al. found in a study with an average follow-up of 21.7 months that patients with metabolic syndrome had a significantly higher recurrence rate after catheter ablation compared to patients without metabolic syndrome (17). In contrast, Choi et al. showed that although diabetes is associated with the recurrence of atrial fibrillation, this association is not independent after adjusting for other cardiovascular risk factors (18). However, these studies mainly focus on chronic metabolic conditions and overlook the potential impact of acute metabolic changes. Our research fills this cognitive gap and demonstrates that stress-induced hyperglycemia—a transient but pathophysiological phenomenon driven by neuroendocrine and inflammatory activation—may be an important determinant of atrial fibrillation recurrence (19–22). SHR provides a more accurate assessment of metabolic stress by simultaneously considering acute and chronic blood glucose control status, which has been validated in prognostic studies of other cardiovascular diseases such as heart failure and acute myocardial infarction (23–25). In our queue, the critical value of SHR 0.91 effectively distinguished patients with different risk levels, and the high SHR group showed a significant association with recurrence, indicating that SHR may become an effective tool for identifying high-risk patients who require more proactive preoperative optimization (26).

The potential pathophysiological mechanisms underlying the association between stress-induced hyperglycemia and atrial fibrillation recurrence deserve further exploration. Acute hyperglycemia may promote atrial electrophysiological remodeling through various pathways: firstly, the hyperglycemic environment enhances oxidative stress, leading to an increase in reactive oxygen species (ROS) production, which in turn damages the ion channel function of myocardial cells (27, 28); Secondly, the systemic inflammatory response induced by hyperglycemia may activate a cascade of pro-inflammatory cytokines, leading to atrial fibrosis and abnormal electrical conduction (29–31); Thirdly, endothelial dysfunction and microvascular injury may lead to inadequate perfusion of atrial tissue, increasing the risk of local ischemia (32). These mechanisms working together may reduce the effectiveness of ablation and promote the recurrence of atrial fibrillation. It is worth noting that our study found that the association between SHR and recurrence of atrial fibrillation is consistent in both diabetes and non diabetes patients, which indicates that stress hyperglycemia may affect the prognosis of atrial fibrillation through general pathophysiological mechanisms, not limited to specific patient groups. This finding is consistent with the recent meta-analysis results, emphasizing the practicability of robust risk predictors in a wide range of people, and also providing important enlightenment for clinicians: even in non diabetes patients, the optimization of preoperative metabolic status may improve the prognosis after ablation (33).

Our research findings have significant clinical implications for atrial fibrillation management strategies. Firstly, as a simple and easily accessible biomarker, SHR can be seamlessly integrated into existing preoperative assessment processes to help identify high-risk patients. For patients with elevated SHR, clinical doctors may consider adopting more proactive preoperative metabolic optimization strategies, such as strengthening blood glucose control, anti-inflammatory treatment, or delaying surgery until metabolic status stabilizes. Secondly, our findings support the comprehensive cardiovascular risk factor intervention strategy confirmed in the ARREST-AF trial, which shows that preoperative adjustment of risk factors can significantly reduce the recurrence rate of atrial fibrillation after ablation. Third, we observed that the SGLT2 inhibitor use rate was low in patients in the relapse group, which suggests that specific anti diabetes drugs may have a cardioprotective effect beyond blood glucose control. SGLT2 inhibitors have been shown to have anti-inflammatory, anti oxidative stress, and improved myocardial energy metabolism effects (34, 35), which may be particularly beneficial for patients with atrial fibrillation. Therefore, SGLT2 inhibitors may be the first choice for patients with atrial fibrillation complicated with diabetes, especially in patients who plan to receive catheter ablation. Finally, our findings emphasize the importance of interdisciplinary collaboration, as collaboration between endocrinologists and arrhythmia specialists may optimize the management of such complex patients.

Our research clearly establishes the independent predictive value of the SHR for prognosis after RFCA in humans for the first time, filling a knowledge gap in this field. This study has several limitations. Its retrospective, single-center design inherently limits causal inference. The assessment of left atrial size relied on linear diameter rather than the more robust volume index (LAVI), which may have reduced the sensitivity of our atrial remodeling assessment. Finally, despite multivariate adjustments, the possibility of unmeasured confounding cannot be entirely excluded. Nevertheless, the relatively large sample size, standardized follow-up, and advanced statistical modeling are key strengths of our analysis.

## 5 Conclusion

In summary, SHR is a reliable and readily available biomarker that is significantly associated with atrial fibrillation recurrence after RFCA. Its predictive value is consistent across diabetic and non-diabetic subgroups, enhancing its potential for broad clinical application. Integrating SHR into pre-procedural risk assessment could facilitate more personalized patient management and ultimately improve long-term outcomes after catheter ablation.

## Data Availability

The raw data supporting the conclusions of this article will be made available by the authors, without undue reservation.

## Glossary

CAD: coronary artery disease
DM: diabetes mellitus
CI: cerebral infarction
COPD: chronic obstructive pulmonary disease
BMI: body mass index
HbA1c: glycated hemoglobin A1c
TC: total cholesterol
TG: triglyceride
HDL-C: high-density lipoprotein cholesterol
LDL-C: low-density lipoprotein cholesterol
UA: uric acid
Cr: creatinine
LAD: left atrial diameter
LVEDD: left ventricular end-diastolic diameter
IVS: interventricular septal thickness
LVPW: left posterior wall thickness
LVEF: left ventricular ejection fraction
SHR: stress hyperglycemia ratio
ACEI + ARB: angiotensin-converting enzyme inhibitors/angiotensin receptor blockers
ABG: admission blood glucose
TSH: thyroid stimulating hormone
AF: atrial fibrillation
RFCA: radiofrequency catheter ablation
ROC: receiver operating characteristic
AUC: area under the curve
RFA: radiofrequency ablation
eAG: estimated average glucose
ABG: admission blood glucose
CRYO: cryoballoon ablation
AAD: antiarrhythmic drug
ACT: activatedclottingtime
PVI: pulmonary vein isolation
CARTO: three-dimensional electroanatomical mapping system
non-PV: non-pulmonary vein
CTI: cavotricuspid isthmus
AT: atrial tachycardias
CIs: confidence intervals
HRs: Hazard Ratios.
