# Supplementary material for: Relationship between the stress–hyperglycemia ratio and atrial fibrillation recurrence risk after radiofrequency catheter ablation: a retrospective study
Source: Front Cardiovasc Med. 2025 Dec 2;12:1699551. doi: 10.3389/fcvm.2025.1699551 (PMC12705545; doi:10.3389/fcvm.2025.1699551)
Supplement: Supplementary file 1 [file Datasheet1.pdf]

Supplementary Table S1. Sensitivity Analysis of the Association Between SHR and AF Recurrence After Excluding Patients with Prior Open-Heart Surgery.

Characteristics	HR(95% CI) Univariate analysis	P value Univariate analysis	HR(95% CI) Multivariate analysis	P value Multivariate analysis
Sex				
Male	Reference			
Female	0.949 (0.667 - 1.351)	0.772		
Age	0.997 (0.980 - 1.014)	0.735		
Alcohol				
NO	Reference		Reference	
YES	1.477 (0.985 - 2.216)	0.059	1.871 (1.223 - 2.863)	0.004
Statins				
NO	Reference			
YES	1.069 (0.754 - 1.516)	0.709		
antiarrhythmics				
YES	Reference		Reference	
NO	1.709 (1.153 - 2.533)	0.008	1.562 (1.044 - 2.338)	0.030
COPD				
NO	Reference			
YES	1.063 (0.620 - 1.823)	0.823		
Anticoagulants				
YES	Reference			
NO	1.407 (0.619 - 3.200)	0.415		
Diabetes				
YES	Reference		Reference	
NO	0.599 (0.422 - 0.850)	0.004	1.364 (0.792 - 2.348)	0.263
Operators	0.959 (0.860 - 1.069)	0.447		

AF type				
Persistent	Reference			
Paroxysmal	0.961 (0.677 - 1.364)	0.824		
AF duration time	1.030 (0.969 - 1.095)	0.350		
SGL-2 inhibitor				
YES	Reference		Reference	
NO	1.434 (0.937 - 2.196)	0.097	1.747 (1.088 - 2.806)	0.021
SHR≥0.91				
NO	Reference		Reference	
YES	3.872 (2.679 - 5.596)	< 0.001	3.451 (2.311 - 5.154)	< 0.001
LA(mm)	1.063 (1.036 - 1.092)	< 0.001	1.031 (1.004 - 1.060)	0.027
LV(mm)	1.025 (0.997 - 1.053)	0.084	1.001 (0.975 - 1.028)	0.946
HbA1c (g/L) (%)	1.471 (1.250 - 1.730)	< 0.001	1.597 (1.250 - 2.041)	< 0.001
BMI	0.966 (0.917 - 1.018)	0.192		
HB(g/dL)	0.997 (0.987 - 1.008)	0.582		
UA(umol/l)	1.002 (1.000 - 1.004)	0.024	1.003 (1.001 - 1.005)	< 0.001
TSH(mg/l)	0.991 (0.952 - 1.031)	0.645		

BMI body mass index, UA uric acid, Cr creatinine, LAD left atrial diameter, LVEDD left ventricular end diastolic diameter,SHR stress hyperglycemia ratio,TSH thyroid stimulating hormone，AF atrial fibrillation, HbA1c glycated hemoglobin A1c, COPD Chronic obstructive pulmonary disease，Hb Hemoglobin.

Supplementary Table S2. Sensitivity Analysis of the Association Between SHR and AF Recurrence Using Tertile Categories

Characteristics	HR(95% CI) Univariate analysis	P value Univariate analysis	HR(95% CI) Multivariate analysis	P value Multivariate analysis
AF type				
Persistent	Reference			
Paroxysmal	0.971 (0.686 - 1.376)	0.870		
sex				
Male	Reference			
Female	0.972 (0.684 - 1.380)	0.872		
age	0.996 (0.979 - 1.014)	0.689		
COPD				
YES	Reference			
NO	0.880 (0.521 - 1.485)	0.632		
Smoking				
YES	Reference			
NO	0.896 (0.609 - 1.317)	0.575		
Drink				
NO	Reference		Reference	
YES	1.461 (0.979 - 2.180)	0.063	1.437 (0.945 - 2.186)	0.090
Statins				
YES	Reference			
NO	0.966 (0.682 - 1.367)	0.844		
Antiarrhythmics				
YES	Reference		Reference	
NO	1.711 (1.156 - 2.534)	0.007	1.668 (1.117 - 2.492)	0.012
Anticoagulants				
YES	Reference			
NO	1.319 (0.580 - 3.003)	0.509		
SGL-2 inhibitor				
NO	Reference		Reference	
YES	0.680 (0.445 - 1.041)	0.076	0.583 (0.358 - 0.949)	0.030
LAD(mm)	1.070 (1.042 - 1.099)	< 0.001	1.039 (1.011 - 1.068)	0.006
LVEDD(mm)	1.025 (0.997 - 1.053)	0.082	0.999 (0.972 - 1.026)	0.931
Operators	0.967 (0.867 - 1.078)	0.547		
HbA1c (%)	1.488 (1.267 - 1.749)	< 0.001	1.384 (1.103 - 1.736)	0.005
BMI	0.967 (0.918 - 1.018)	0.202		
Hemoglobin, g/dL	0.996 (0.986 - 1.007)	0.506		
UA(umol/l)	0.998 (0.996 - 1.000)	0.034	1.000 (0.998 - 1.002)	0.832
TSH(mg/l)	1.005 (0.975 - 1.037)	0.733		

Diabetes mellitus				
NO	Reference		Reference	
YES	1.735 (1.225 - 2.458)	0.002	1.010 (0.594 - 1.720)	0.970
SHR-group				
1	Reference		Reference	
2	1.522 (0.878 - 2.641)	0.135	1.451 (0.832 - 2.529)	0.189
3	6.262 (3.844 - 10.200)	< 0.001	5.025 (3.015 - 8.375)	< 0.001

COPD Chronic obstructive pulmonary disease， BMI body mass index, UA uric acid,, LAD left atrial diameter, LVEDD left ventricular end diastolic diameter, SHR stress hyperglycemia ratio, TSH thyroid stimulating hormone， AF:atrial fibrillation
